# Reductive soil disinfestation alleviates continuous cropping obstacles in tobacco by reshaping microbial communities and improving soil properties in karst regions

**DOI:** 10.3389/fmicb.2026.1841207

**Published:** 2026-05-28

**Authors:** Mengjiao Ding, Nianjie Shang, Bo Gong, Qingli Liu, Caibin Li, Erwei Zhao, Yanling Zhang, Taibo Liang

**Affiliations:** 1 Zhengzhou Tobacco Research Institute of CNTC, Zhengzhou, China; 2 College of Tobacco Science of Guizhou University, Guiyang, China; 3 Guizhou Provincial Key Laboratory for Tobacco Quality, College of Tobacco Science, Guizhou University, Guiyang, China; 4 Institute of Crop Germplasm Resources, Guizhou Academy of Agricultural Sciences, Guiyang, China; 5 Institute of Agricultural Resources and Regional Planning, Chinese Academy of Agricultural Sciences, Beijing, China; 6 Bijie Branch of Guizhou Provincial Tobacco Company, Bijie, China

**Keywords:** continuous cropping obstacles, disease control, microbial community, reductive soil disinfestation, soil health

## Abstract

Reductive soil disinfestation (RSD) shows potential for alleviating continuous cropping obstacles in greenhouse agriculture. However, its effects remain unclear in dryland tobacco cropping systems. A field experiment was conducted in continuous cropping tobacco fields in Bijie City, Guizhou Province. The study evaluated RSD treatments with different amounts of organic material (0, 140, and 280 kg per 667 m^2^) combined with a 20% reduction in chemical fertilizer. Soil physicochemical properties, microbial community structure, tobacco growth, and disease control were assessed. RSD treatments significantly increased soil pH and available nutrient levels. These improvement effects persisted until the tobacco maturation stage. RSD treatments significantly reshaped soil microbial communities, increasing fungal diversity while reducing the abundances of soil-borne pathogens such as *Fusarium* and *Gibberella*. RSD promoted the proliferation of beneficial functional groups. These included *Acidobacteriota, Chloroflexi*, and *Bacillus*. Functional predictions indicated a decline in plant pathogenic fungi. Conversely, saprophytic fungi and nitrogen-cycling bacteria functions were enhanced. The 280 kg treatment (T3) performed best, reducing bacterial wilt incidence by 93.3% and tobacco mosaic virus incidence by 76.5%, while improving leaf potassium-to-chlorine ratio. The treatment with 140 kg organic material (T2) showed weaker effects. RSD with high organic material input is an effective green strategy for managing continuous cropping obstacles in karst tobacco regions.

## 1 Introduction

Continuous cropping obstacles represent a major constraint on tobacco production worldwide (Yu et al., 2023). Prolonged monoculture typically leads to soil acidification, nutrient imbalance, decline in organic matter, and accumulation of soilborne pathogens, all of which reduce crop growth and leaf quality and ultimately cause economic losses (Chen et al., 2023). Organic matter declines and pH levels drop under continuous cropping (Shen et al., 2020). In China, continuous tobacco cropping is widely practiced due to limited arable land and sustained market demand (Liu et al., 2024). Bijie City in Guizhou Province, situated in a typical karst mountain area with shallow and acidic yellow soils, exemplifies the severity of this problem (Guo et al., 2022). The fragile ecosystem there further exacerbates the negative effects of continuous cropping, threatening the sustainability of local tobacco production (Chen et al., 2024; Wang et al., 2022).

Reductive soil disinfestation (RSD) has emerged as an environmentally friendly green restoration technology. It was first used in the United States to replace chemical fumigants (Zhu et al., 2023). This technique involves incorporating easily decomposable organic materials into the soil, followed by flooding and airtight film covering to create a highly reductive anaerobic environment. Pathogens are efficiently inactivated. Soil physicochemical properties are simultaneously improved. Microbial community structures are effectively reshaped (Huang et al., 2019). In recent years, RSD technology has been widely applied in managing continuous cropping obstacles for greenhouse vegetables and fruits in China, demonstrating excellent control efficacy against soilborne diseases such as bacterial wilt, root rot, and root-knot nematode disease (Liu et al., 2018). RSD is an ideal method for addressing multi-factor continuous cropping obstacles (Wu et al., 2024a). However, its applicability and optimal parameters in upland tobacco systems, especially in karst regions with yellow acidic soils, remain largely unexplored.

We hypothesized that RSD integrated with organic amendment and reduced chemical fertilization could synergistically improve soil health, restructure microbial networks, suppress soil-borne pathogens, and thereby alleviate continuous cropping obstacles in tobacco. To test this hypothesis, we conducted a field experiment in Bijie City with different amounts of organic material and reduction in chemical fertilizer. This study aims to determine the optimal RSD application scheme for continuously cropped tobacco fields in Bijie. Specifically, it evaluates the scheme's impacts on soil physicochemical properties, microbial community assembly, tobacco agronomic performance, and disease control. The findings are expected to provide a scientific foundation for green soil remediation and sustainable tobacco production in karst mountainous areas.

## 2 Materials and methods

### 2.1 Study site

The field experiment was conducted from April to September 2023 in the Qixingguan Experimental Zone of Bijie City, Guizhou Province (105°19′E, 27°21′N). This region features typical karst landforms, with yellow soils and limestone soils as the predominant types. The average altitude is 1,511 m, the mean annual temperature is 12.5 °C, and the frost-free period lasts 250 days. The previous crop was tobacco, which had been continuously cropped for at least seven years, resulting in severe soilborne disease occurrence. The baseline physicochemical properties of the experimental soil were as follows: pH 5.42, soil organic matter (SOM) 18.6 g·kg^−1^, alkali-hydrolyzable nitrogen (AN) 112.4 mg·kg^−1^, available phosphorus (AP) 18.7 mg·kg^−1^, and available potassium (AK) 156.3 mg·kg^−1^.

### 2.2 Experimental materials

The tobacco cultivar used was “Anyan 3” (*Nicotiana tabacum* L.), provided by the Bijie Tobacco Company. The organic material was a mixture of distiller's grains and corn straw at a dry weight ratio of 3:7. The corn straw was air-dried and crushed to pass through a 2 cm sieve. The basic properties of the mixture were as follows: total carbon 270.57 g·kg^−1^, total nitrogen 10.72 g·kg^−1^, and a carbon-to-nitrogen ratio of 25.24. The fertilizers used were a specialized tobacco compound fertilizer (N:P_2_O5:K_2_O = 10:10:25, used as basal fertilizer) and potassium nitrate (N 13.5%, K_2_O 44.5%, used as topdressing).

### 2.3 Experimental design

A single-factor completely randomized block design was employed with four treatments and three replicates. Each plot covered 30 m^2^ and was separated by 50 cm buffer strips. Protection rows were established around the perimeter. Treatment details are provided in Table 1. The reduced fertilization treatment involved a 20% reduction relative to conventional rates.

**Table 1:** Experimental design of reductive soil disinfestation (RSD) treatments.

Treatment	Fertilizer application	Anaerobic condition	Organic material (kg/667 m^2^)
CK	Conventional	–	–
T1	−20%	Flooding + plastic mulching	–
T2	−20%	Flooding + plastic mulching	140
T3	−20%	Flooding + plastic mulching	280

RSD treatment was implemented 20 days before tobacco transplanting. Organic materials were applied uniformly to the soil surface on April 10, 2023, and mixed via rotary tillage. The soil was irrigated to 85% of field capacity. Plots were immediately sealed with 0.08 mm thick transparent polyethylene film. This flooded and sealed state was maintained for 20 days. After uncovering the film, the plots were allowed to drain naturally. Tobacco seedlings were transplanted when the soil moisture content reached 60%. The transplanting row spacing was 1.1 m, and the plant spacing was 0.5 m. All other field management practices were consistent across treatments.

### 2.4 Sample collection

Soil samples were collected from the 0–20 cm plow layer at 0, 30, 60, 90, and 120 days after RSD treatment. A five-point sampling method was used to collect soil from each plot (Chen et al., 2024). Impurities were removed before the soil was homogenized via the quartering method and placed in sterile bags. Soil samples were collected at 0, 30, 60, 90, and 120 days after RSD treatment. Two time points (0 d and 60 d) were selected for microbial community analysis. The 0 d sample reflected the immediate effect of RSD treatment, while 60 d corresponded to the vigorous growth stage of tobacco, when plant-microbe interactions and disease pressure typically peak. Samples from these two time points were split into two subsamples. One subsample was stored at −80 °C for high-throughput sequencing, and the other was air-dried and sieved through 2 mm and 0.25 mm meshes for physicochemical analysis. The remaining time points (30, 90, 120 d) were used exclusively for monitoring soil physicochemical properties only.

### 2.5 Soil physicochemical property analysis

Soil pH was determined using the potentiometric method with a water-to-soil ratio of 2.5–1 (Bao, 2000). Soil organic matter was measured using the potassium dichromate oxidation and external heating method (Alary et al., 2013). Alkali-hydrolyzable nitrogen was determined by the alkaline hydrolysis diffusion method (Feng et al., 2025). Available phosphorus was extracted with 0.5 mol·L^−1^ sodium bicarbonate and measured using molybdenum-antimony colorimetry (Huang et al., 2024). Available potassium was extracted with 1 mol·L^−1^ ammonium acetate and analyzed via flame photometry (Shao et al., 2020).

### 2.6 Soil Microbial community structure

Total genomic DNA was extracted from 0.5 g of fresh soil using the FastDNA^®^ Spin Kit for Soil (MP Biomedicals, USA, Cat. No. 116560200). DNA integrity was verified using 1% agarose gel electrophoresis. Concentration and purity were assessed with a NanoDrop 2000 spectrophotometer (Thermo Scientific, USA) (Idbella et al., 2024). The V3–V4 region of bacterial 16S rRNA gene was amplified with primers 338F (5′-ACTCCTACGGGAGGCAGCAG-3′) and 806R (5′-GGACTACHVGGGTWTCTAAT-3′). The fungal ITS1 region was amplified with primers ITS1F (5′-CTTGGTCATTTAGAGGAAGTAA-3′) and ITS2R (5′-GCTGCGTTCTTCATCGATGC-3′) (Li et al., 2023; Wang et al., 2026). Raw data were filtered using Trimmomatic v0.33 (Duan et al., 2025). Primers were removed with Cutadapt v1.9.1 (Nelkner et al., 2019). Sequences were assembled using USEARCH v10 (Edgar, 2010) and chimeras were removed with UCHIME v8.1 (Edgar et al., 2011). Operational taxonomic units (OTUs) were clustered at 97% similarity. OTUs with an abundance below 0.005% were excluded (Li et al., 2023). Taxonomic classification was performed using the QIIME2 Naive Bayes classifier and the SILVA database with a 70% confidence threshold (Bolyen et al., 2019). Alpha diversity indices including Chao1, Shannon, and Simpson were calculated from the OTU table (Bokulich et al., 2013). Beta diversity was evaluated through Principal Coordinate Analysis (PCoA) based on Bray-Curtis distances (Lozupone and Knight, 2005). Significant differences between groups were identified using Linear Discriminant Analysis Effect Size (LEfSe) with an LDA threshold >2.0 (Segata et al., 2011). Bacterial and fungal functions were predicted using FAPROTAX (version 1.2.6) and FUNGuild (version 1.0), respectively (Louca et al., 2016; Nguyen et al., 2016).

### 2.7 Agronomic traits and disease investigation

Agronomic traits were measured according to the Chinese tobacco industry standard YC/T 142-2010 (Investigating and measuring methods of agronomical character of tobacco) (State Tobacco Monopoly Administration, 2010). Plant height, stem girth, and leaf area were recorded at each growth stage. Disease incidence and severity indices were investigated following the national standard of China GB/T 23222-2008 (Grade and investigation method of tobacco diseases and insect pests) (Standardization Administration of China, 2008).

### 2.8 Chemical composition of tobacco leaves

Leaf samples of the C3F grade were dried at 60 °C to constant weight and ground to pass a 0.25 mm sieve. Contents of total soluble sugar, reducing sugar, and starch were determined using the 3,5-dinitrosalicylic acid colorimetric method. Nicotine content was analyzed by UV spectrophotometry. Total nitrogen was measured via the Kjeldahl method. Potassium was analyzed by flame photometry. Chlorine was determined using silver nitrate titration. These procedures followed the methods described by (Han 2014).

### 2.9 Statistical analysis

Data for soil physicochemical properties, tobacco agronomic traits, disease indices, economic traits, and chemical components were compiled using Excel 2019. Differences among treatments were assessed by one-way analysis of variance (ANOVA), followed by Duncan's multiple range test for *post-hoc* comparisons, with the significance level set at α = 0.05. Statistical analysis and graphing were performed using Origin 2021. Microbial diversity data analyses and differential testing were conducted using the Majorbio Cloud Platform (https://v.majorbio.com/project-center/) and R software (v4.3.1). Mantel tests and Pearson correlation analyses were employed to explore the relationships between microbial community structure and soil physicochemical factors.

## 3 Results

### 3.1 Effects of RSD treatment on physicochemical properties

**Figure 1:**
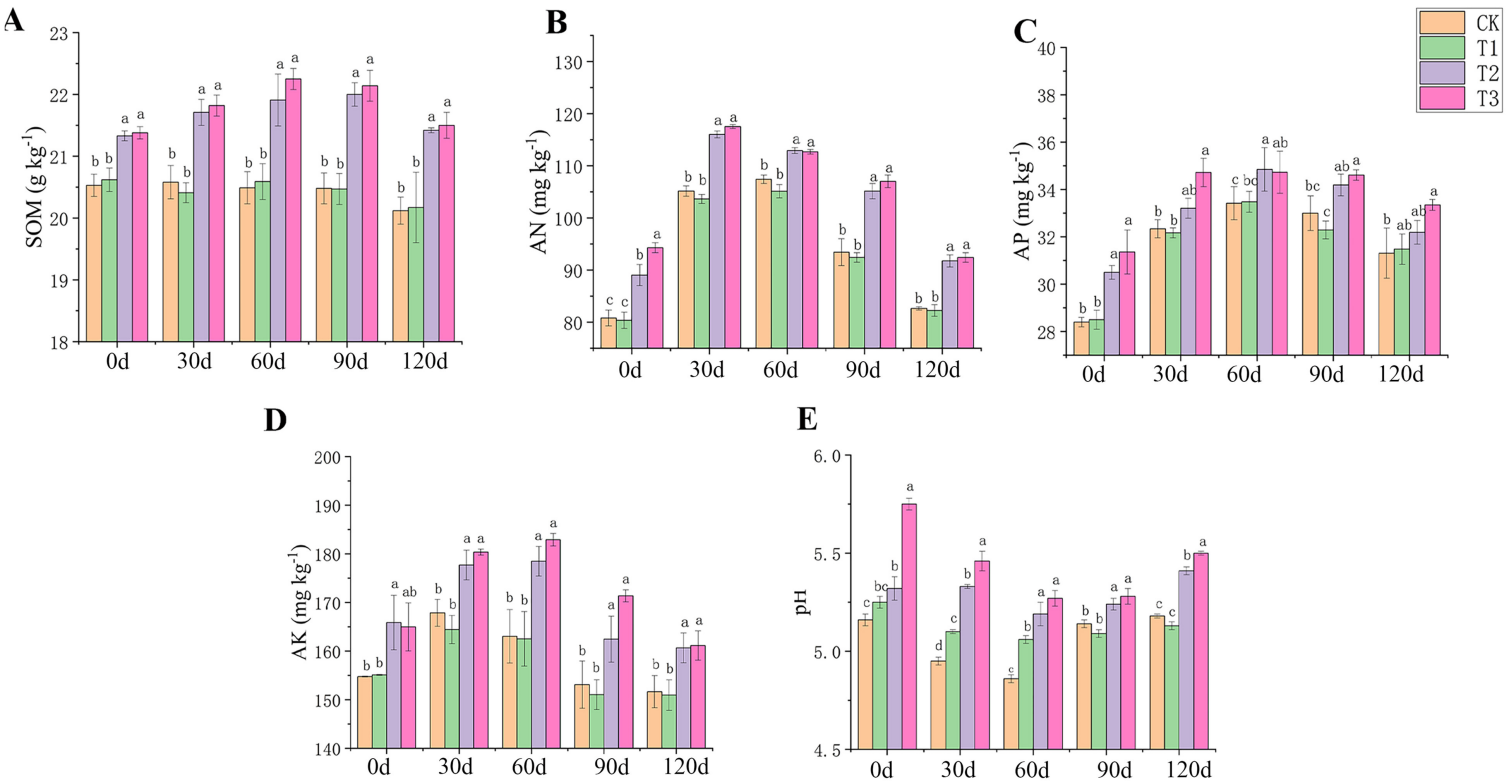
Soil[0mm][-8mm]Q10 physicochemical properties across different treatment groups. **(A)** Soil organic matter (SOM); **(B)** Alkali-hydrolyzable nitrogen (AN); **(C)** Available phosphorus (AP); **(D)** Available potassium (AK); **(E)** Soil pH. Data are presented as means ± standard error (*n* = 3). Different lowercase letters above the bars indicate significant differences among treatments at each sampling time point (*P* < 0.05, Duncan's multiple range test).

RSD treatment significantly improved the physicochemical properties of the continuously cropped soil. Immediately following the RSD treatment period, compared with the CK group, SOM, AN, AP, AK, and pH were significantly higher in the T2 and T3 treatments (Figure 1). Specifically, T2 and T3 enhanced soil fertility, increasing SOM, AN, AP, and AK by 3.90%, 10.18%, 7.39%, and 7.17%, and by 4.14%, 16.69%, 10.42%, and 6.60%, respectively. Concurrently, soil pH rose by 0.16 and 0.59 units. No significant differences in these parameters were observed between the T1 treatment and CK. The soil improvement effects from the T2 and T3 treatments persisted throughout the entire tobacco growth period (30-120 d post-transplanting). Compared to CK, the T2 and T3 treatments sustained average increases in SOM and AN ranging from 5.49% to 8.59% and 4.91% to 14.55%, respectively. Average increases in AP and AK ranged from 2.69% to 7.36% and 5.86% to 12.19%, respectively. Soil pH values were also continuously improved, with average increases of 0.10–0.51.

### 3.2 Soil microbial alpha diversity analysis

RSD treatment significantly altered soil microbial alpha diversity. Immediately after RSD treatment (0 d), the T2 and T3 treatments increased the fungal Chao1 index by 7.41% and 18.89%, respectively, and the Simpson index by 33.90% and 16.27% compared with CK (Table 2). This indicated that RSD with organic material addition enhanced both the richness and diversity of the soil fungal community. The promoting effect on fungal diversity was more pronounced in the T2 treatment. The bacterial community exhibited a different response pattern. The Simpson index for bacteria in the T2 and T3 treatments decreased by 17.07% and 34.15% compared with CK, indicating reduced diversity. The decrease in the T3 treatment was statistically significant. The bacterial Chao1 index was significantly higher in the T2 treatment than in CK. This suggests that RSD treatment increased bacterial richness while simultaneously decreasing its evenness, exerting a selective enrichment effect on the bacterial community.

**Table 2:** Analysis of variance (ANOVA) for fungal and bacterial alpha diversity indices under different RSD treatments.

Growth stage	Treatment	Fungi		Bacteria	
		Chao1 index	Simpson index	Chao1 index	Simpson index
Post-RSD	CK	937.95 ± 36.65 a	0.0295 ± 0.0029 a	4,867.89 ± 94.69 bc	0.0041 ± 0.0004 a
	T1	958.17 ± 21.59 a	0.0292 ± 0.0029 a	5,382.11 ± 59.57 a	0.0033 ± 0.0001 ab
	T2	1,007.46 ± 96.41 a	0.0395 ± 0.0024 a	5,075.33 ± 72.11 b	0.0034 ± 0.0001 ab
	T3	1,115.16 ± 39.60 a	0.0343 ± 0.0145 a	4,805.47 ± 22.33 c	0.0027 ± 0.0004 b
Vigorous growing	CK	847.68 ± 42.12 a	0.0398 ± 0.0389 a	3,898.47 ± 163.97 b	0.0076 ± 0.0008 a
	T1	824.32 ± 50.66 a	0.1077 ± 0.0721 a	4,359.49 ± 120.23 ab	0.0073 ± 0.0011 a
	T2	916.37 ± 38.30 a	0.0983 ± 0.0642 a	4,680.96 ± 398.07 a	0.0056 ± 0.0020 a
	T3	901.16 ± 55.97 a	0.0957 ± 0.0236 a	4,856.14 ± 142.84 a	0.0043 ± 0.0005 a

Values are means ± standard error (*n* = 3). Different lowercase letters within the same column and growth stage indicate significant differences among treatments at *P* < 0.05. Post-RSD: samples collected immediately after RSD treatment (0 d). Vigorous growth: samples collected at the tobacco vigorous growth stage (60 d).

### 3.3 Analysis of soil microbial community composition

**Figure 2:**
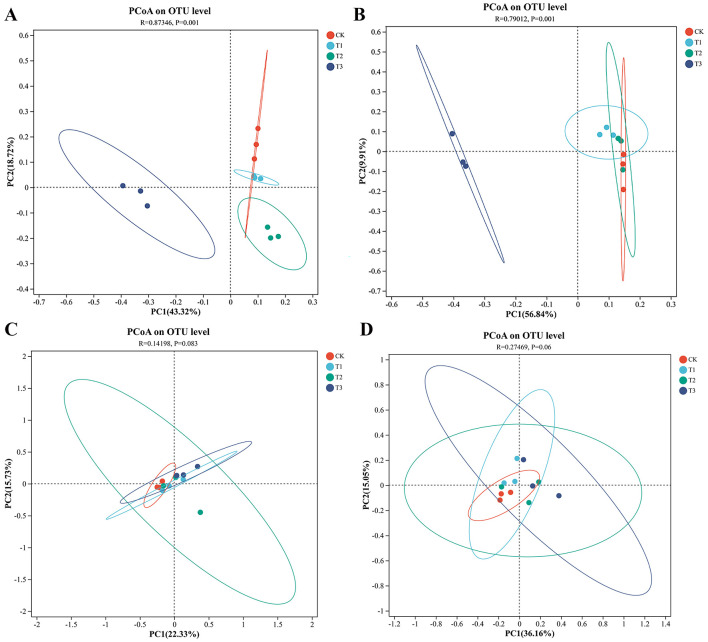
Principal Coordinates Analysis (PCoA) of soil microbial communities based on Bray-Curtis distance. Panels **(A)** and **(B)** show the fungal and bacterial communities, respectively, in soil samples collected immediately after RSD treatment (0 d). Panels **(C)** and **(D)** show the fungal and bacterial communities, respectively, in soil samples collected at the tobacco vigorous growth stage (60 d post-transplanting). Each point represents an individual sample, with different colors and shapes denoting distinct treatment groups. Ellipses represent 95% confidence intervals surrounding the group centroids.

Principal Coordinate Analysis (PCoA) demonstrated that RSD treatment significantly altered the soil microbial community structure (Figure 2). Fungal and bacterial communities in T2 and T3 were significantly separated from CK and T1 after treatment (Figures 2A, B). Fungal community structures became similar across treatments by the vigorous growth stage of tobacco (Figure 2C). In contrast, the bacterial community in T3 remained significantly separated from other treatments (Figure 2D).

**Figure 3:**
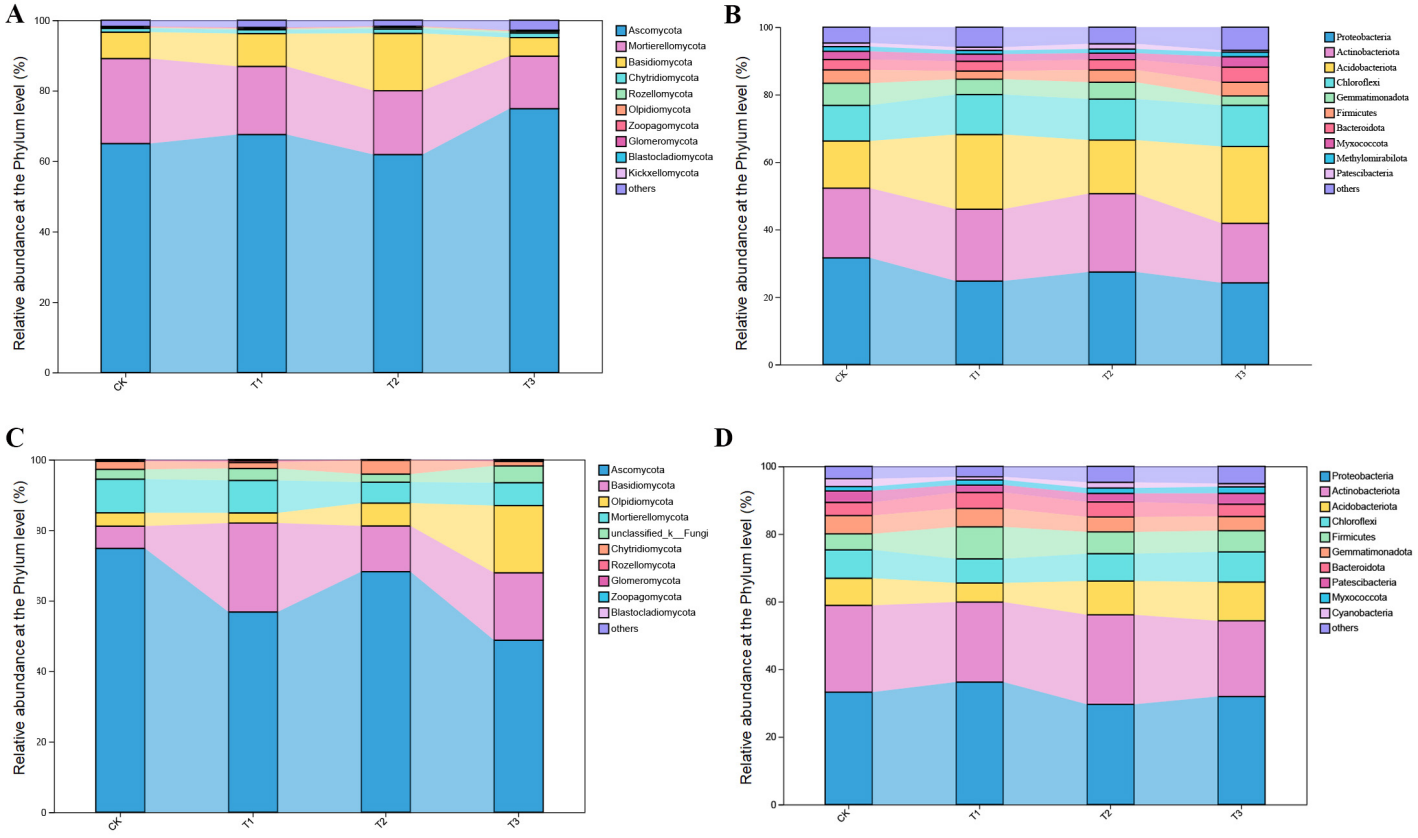
Soil microbial community composition at the phylum levels. Panels **(A–D)** show community composition at the phylum level. Panels **(A)** and **(B)** represent fungal and bacterial communities, respectively, in soil samples collected immediately after RSD treatment (0 d). Panels **(C)** and **(D)** represent fungal and bacterial communities, respectively, in soil samples collected at the tobacco vigorous growing stage (60 d post-transplanting).

At the phylum level, RSD treatment significantly modified microbial community composition. Ascomycota was the dominant fungal phylum across all samples, with relative abundances ranging from 48.80% to 74.89% (Figure 3A). Immediately after RSD treatment (0 d), compared to CK, the relative abundance of Basidiomycota significantly increased by 210% and 120% in the T2 and T3 treatments, respectively. Conversely, the relative abundance of Mortierellomycota decreased by 38.2% and 50.1% in these treatments. At 60 d, the relative abundances of Ascomycota and Mortierellomycota were significantly lower in the T2 and T3 treatments compared to CK (Figure 3C). In contrast, the relative abundances of Basidiomycota and Olpidiomycota were significantly higher. Notably, Olpidiomycota abundance in the T2 and T3 treatments increased by 400% and 410% respectively compared to CK, becoming the second most dominant phylum after Ascomycota. At the bacterial phylum level, Proteobacteria was dominant across all samples (relative abundance 24.2%−36.2%), followed by Actinobacteriota, Acidobacteriota, and Chloroflexi (Figure 3B). Immediately after RSD treatment (0 d), compared to CK, the relative abundance of Proteobacteria decreased by 5.7% and 8.2% in the T2 and T3 treatments, respectively, while Chloroflexi increased by 32.1% and 35.7%. In the T3 treatment, Acidobacteriota and Bacteroidota abundances increased by 62.8% and 47.1% compared to CK, whereas Actinobacteriota decreased by 22.6%. At 60 d, the relative abundance of Proteobacteria remained lower in the T2 and T3 treatments than in CK (decreases of 4.8% and 7.3%, respectively) (Figure 3D). Acidobacteriota maintained its dominant position, with abundances 17.3% and 31.8% higher than CK in the T2 and T3 treatments. Bacteroidota also maintained a high relative abundance in the T3 treatment.

**Figure 4:**
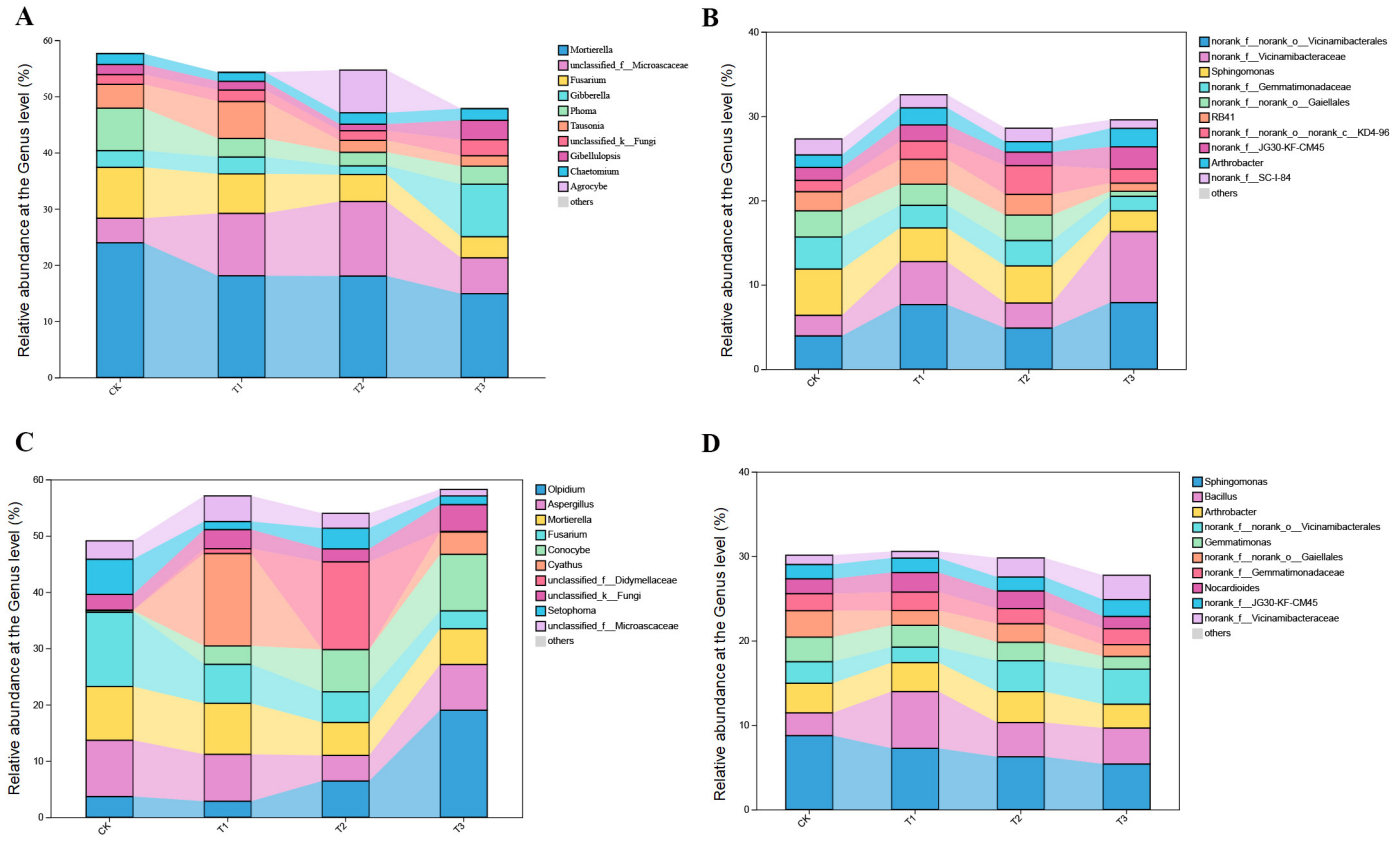
Soil microbial community composition at the genus levels. Panels **(A–D)** show community composition at the genus level. Panels **(A)** and **(B)** represent fungal and bacterial communities, respectively, at 0 d post-RSD, while panels **(C)** and **(D)** represent fungal and bacterial communities, respectively, at 60 d post-transplanting.

At the genus level, several pathogenic fungi were significantly inhibited. Immediately after RSD treatment (0 d), compared to CK, the relative abundances of *Fusarium, Gibberella*, and *Phoma* in the T2 and T3 treatments were reduced by 55.3% and 68.1%, 73.2% and 81.5%, and 48.6% and 62.3%, respectively (Figure 4A). At 60 d, the relative abundances of *Fusarium* in the T2 and T3 treatments remained 32.7% and 41.5% lower than in CK (Figure 4C). Similarly, *Aspergillus* abundances were reduced by 32.1% and 39.8%, and *Mortierella* abundances by 21.3% and 32.6%. Concurrently, the relative abundance of *Olpidium* in the T2 and T3 treatments was 420% and 450% higher than in CK, establishing it as one of the dominant genera in the fungal community during the vigorous growth period. At the bacterial genus level, *Sphingomonas* was a common dominant genus across all treatments (Figure 4B). Immediately after RSD treatment (0 d), compared to CK, the relative abundance of *Sphingomonas* decreased by 18.6%, 28.9%, and 35.1% in the T1, T2, and T3 treatments, respectively. The relative abundances of uncultured dominant genera within *Acidobacteriota* increased by 35.8% and 62.3% in the T2 and T3 treatments, while those within *Gemmatimonadota* increased by 28.6% and 45.2%. During the vigorous growth period, compared to CK, the relative abundance of *Bacillus* was significantly higher in the T2 and T3 treatments, with increases of 45.8% and 62.3%, respectively (*P* < 0.05) (Figure 4D). The uncultured dominant genus within *Acidobacteriota* also maintained a significant abundance advantage, with increases of 21.6% and 38.4% compared to CK (*P* < 0.05).

Linear discriminant analysis Effect Size (LEfSe) identified genus-level fungal biomarkers specific to different RSD treatments in continuously cropped tobacco soils (LDA > 2, *P* < 0.05). Immediately after RSD treatment, CK group was characterized by the soilborne pathogen *Phoma* (Supplementary Figure S1). T2 enriched *Fusarium*, whereas the T3 enriched multiple saprotrophic genera including *Peziza, Exophiala, Tausonia*, and *Gibellulopsis*. At 60 days, CK and T1 still lacked stable biomarkers. T2 was characterized by *Leucoagaricus*, and T3 enriched genera with decomposition and biocontrol potential such as *Myceliophthora* and *Microdochium*. For bacteria, only the organic-amended T2 and T3 treatments harbored specifically enriched taxa. Core biomarkers in the T3 treatment included *Arthrobacter* and uncultured genera within the strictly anaerobic class *Anaerolineae*. The T2 treatment enriched facultative anaerobic groups such as *Gemmatimonas*. No consistent differentially abundant biomarkers were detected in the unamended CK and T1 treatments. At the tobacco vigorous growth stage, the community biomarkers exhibited a clear successional shift. The T3 treatment significantly enriched *Ensifer* and *Microvirga* from the family *Rhizobiaceae*, along with *Fictibacillus*. The T2 treatment enriched *Bryobacter*, uncultured genera from the family *Chitinophagaceae*, and *Cellulomonas*. The CK and T1 treatments still exhibited no consistent enrichment of plant growth-promoting or biocontrol taxa.

### 3.4 Correlation analysis of microbial communities and soil properties

**Figure 5:**
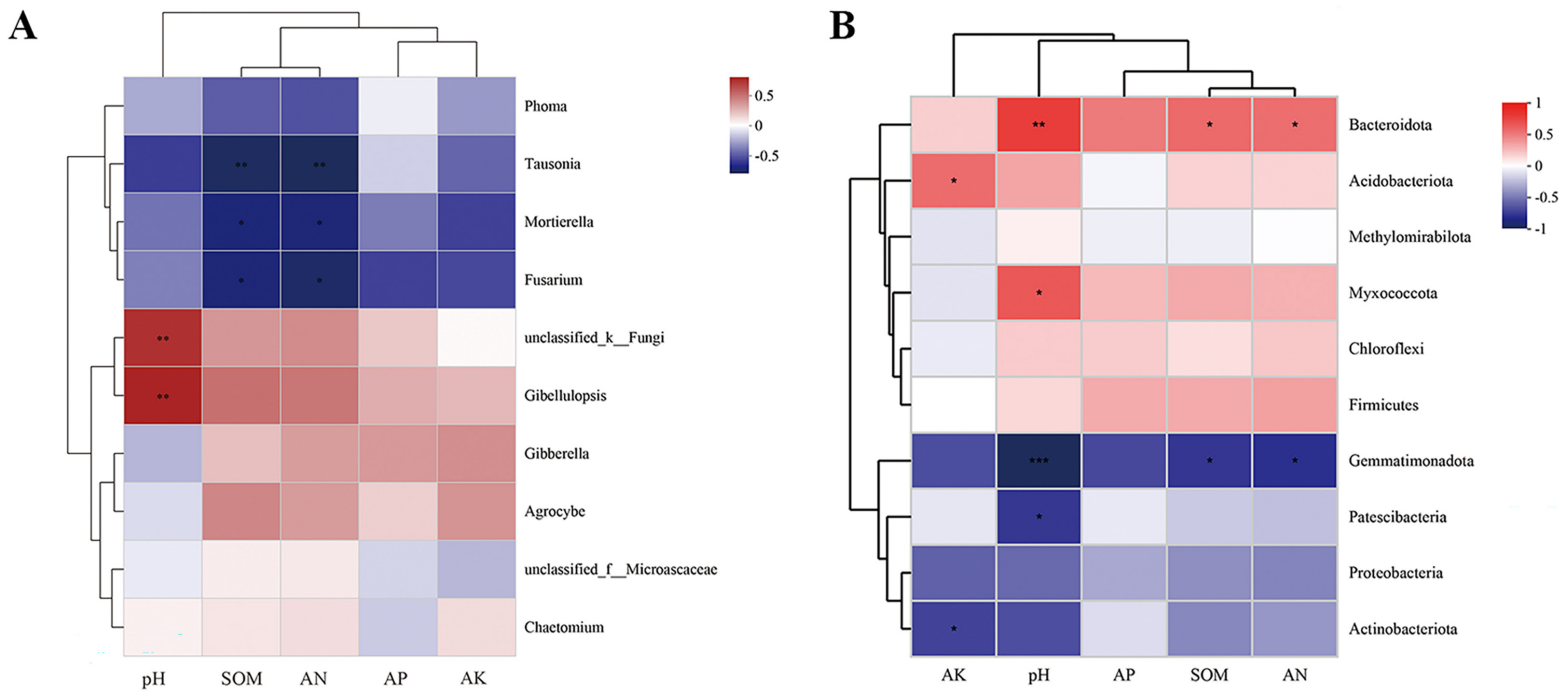
Heatmap of Pearson correlation analysis between soil physicochemical properties and microbial communities after RSD treatment. Panels **(A)** and **(B)** represent fungal and bacterial communities, respectively. *P < 0.05, **P < 0.01, ***P < 0.001. SOM, soil organic matter; AN, alkali-hydrolyzable nitrogen; AP, available phosphorus; AK, available potassium.

Pearson correlation analysis indicated that soil pH, AN, and SOM were the core factors driving microbial community succession. In fungal communities, the relative abundances of *Mortierella, Fusarium*, and *Tausonia* were significantly negatively correlated with soil AN and SOM (*P* < 0.05) (Figure 5A). The abundance of *Gibellulopsis* showed a highly significant positive correlation with soil pH (*P* < 0.01). In bacterial communities, the abundance of *Gemmatimonadota* was significantly negatively correlated with AN and SOM (Figure 5B). In contrast, *Bacteroidota* was significantly positively correlated with pH, AN, and SOM (*P* < 0.05). *Myxococcota* was significantly positively correlated with pH (*P* < 0.05).

**Figure 6:**
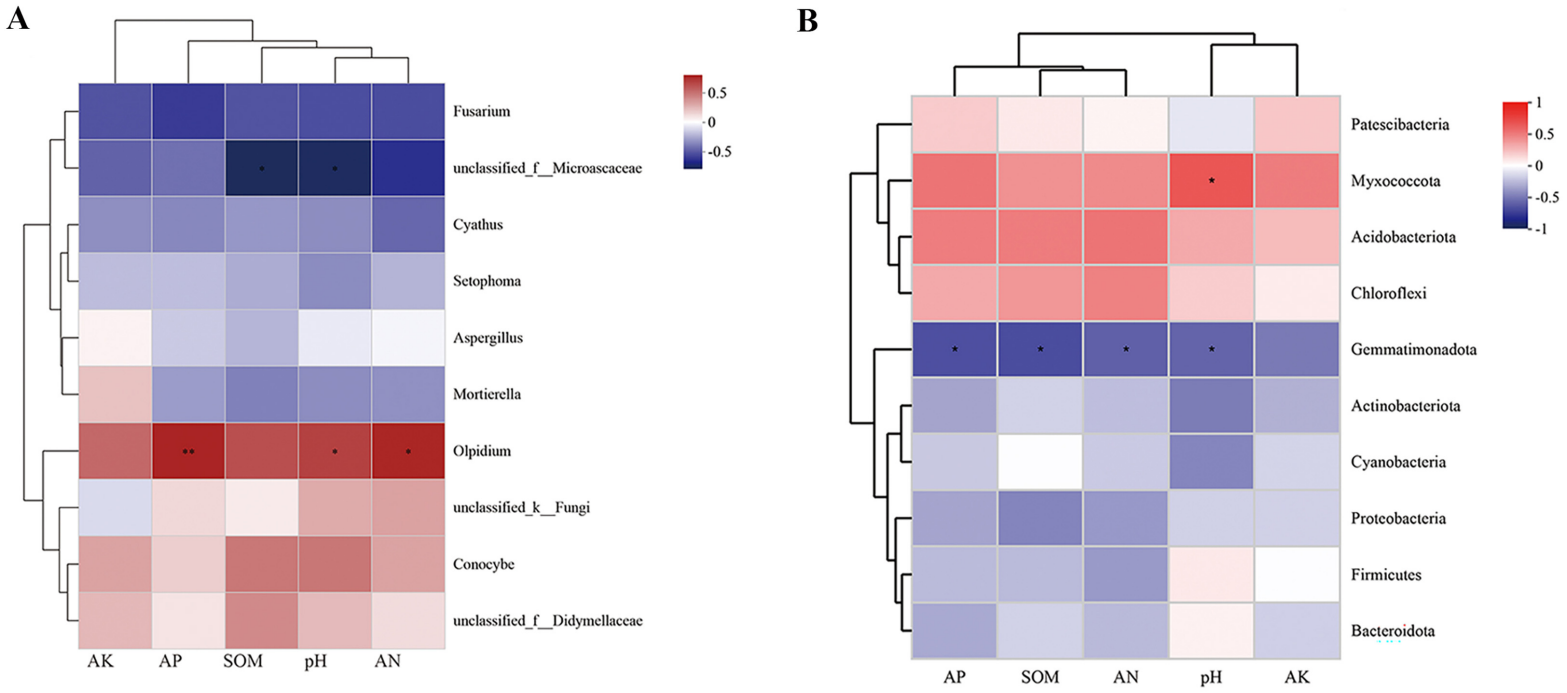
Heatmaps showing Pearson correlation coefficients between soil physicochemical properties and microbial communities at the tobacco vigorous growing stage (60 days after transplanting). Panels **(A)** and **(B)** represent fungal and bacterial communities, respectively. *P < 0.05, **P < 0.01. SOM, soil organic matter; AN, alkali-hydrolyzable nitrogen; AP, available phosphorus; AK, available potassium.

At 60 days, pH, AN, SOM, AP, and AK remained the core drivers of community structure. In fungal communities, the abundance of *Olpidium* was significantly positively correlated with pH, AP, and AN (*P* < 0.05) (Figure 6A). For bacteria, the relative abundance of *Myxococcota* was significantly negatively correlated with soil AK (*P* < 0.05), while the relative abundance of *Gemmatimonadota* remained significantly negatively correlated with soil pH, AN, and SOM (*P* < 0.05) (Figure 6B).

### 3.5 Microbial co-occurrence network analysis

**Figure 7:**
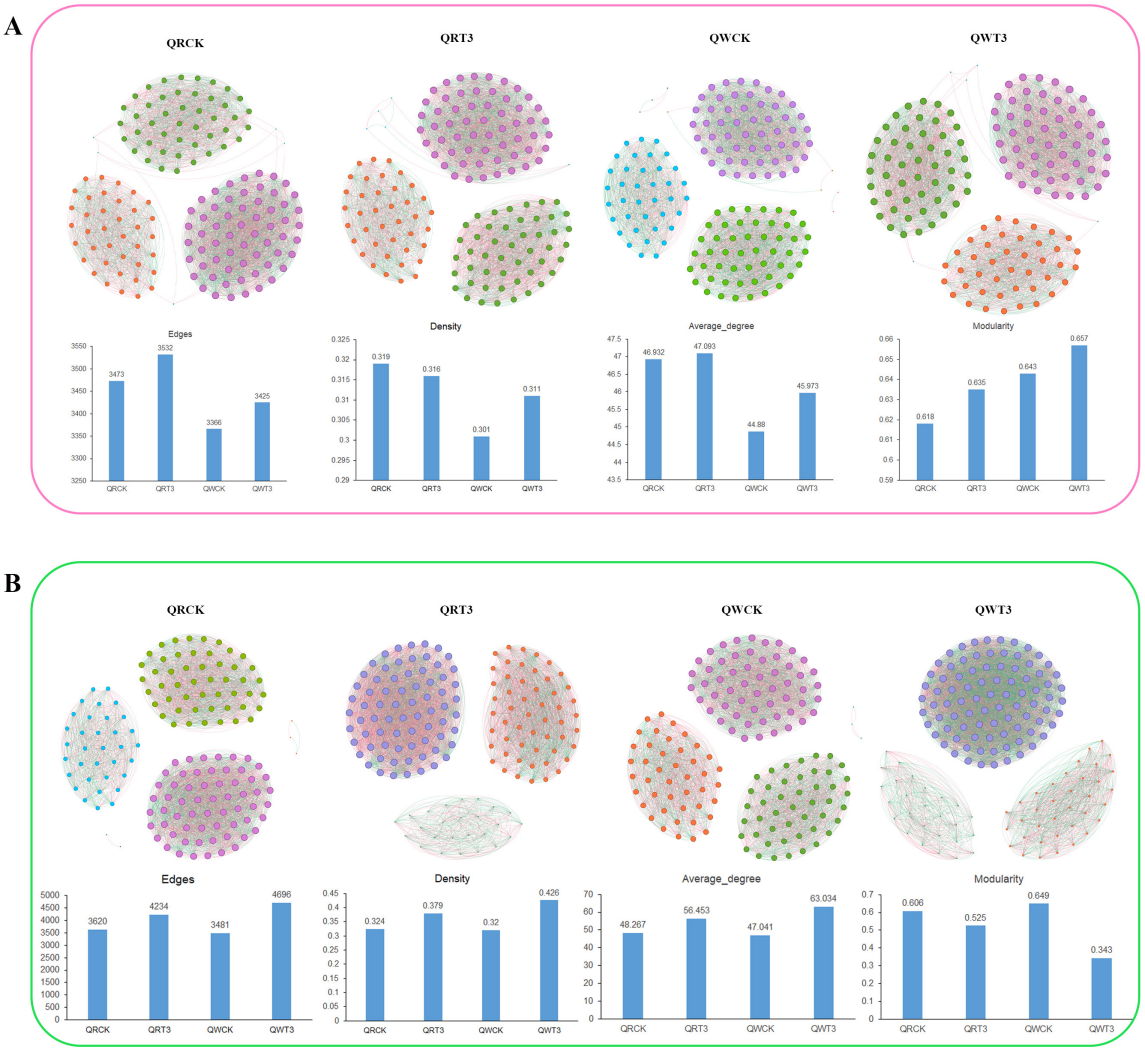
Co-occurrence networks and topological parameters of soil fungal **(A)** and bacterial **(B)** communities. QRCK and QRT3 represent CK and T3 treatments at 0 days after RSD. QWCK and QWT3 represent CK and T3 treatments at 60 days after transplanting. All networks were constructed at the genus level with Spearman correlation (|r| > 0.7, *P* < 0.05).

To elucidate the impact of RSD on soil microbiome interactions, genus-level co-occurrence networks were constructed using Spearman's correlation (|r|>0.7, *P* < 0.05). Topological parameters were calculated to assess structural changes (Figure 7). Fungal networks exhibited notable structural stasis across treatments. At 0 days after RSD treatment, T3 yielded edge counts of 3,532 and an average degree of 47.09, values nearly identical to those of CK (3,473 and 46.93) (Figure 7A). Density and modularity remained statistically indistinguishable between groups. This pattern persisted at 60 days after transplanting, where T3 showed subtle increases in connectivity metrics yet maintained comparable modularity. These observations indicate that RSD did not perturb the fundamental architecture of fungal co-occurrence networks, despite concurrent shifts in alpha diversity. In contrast, bacterial networks were strongly reshaped by RSD treatment. Immediately following treatment, T3 enhanced network complexity, increasing edge number by approximately 600 and elevating average degree from 48.27 to 56.45, while modularity declined from 0.606 to 0.525 (Figure 7B). At 60 days after transplanting, this divergence intensified. T3 harbored over 1200 more edges than CK and achieved a markedly higher average degree (63.03 vs. 47.04), coinciding with a drastic reduction in modularity (0.343 vs. 0.649). Our result demonstrated that RSD promotes bacterial network integration, shifting the community toward a less compartmentalized and more cooperative configuration.

### 3.6 Analysis of soil microbial functional changes

**Figure 8:**
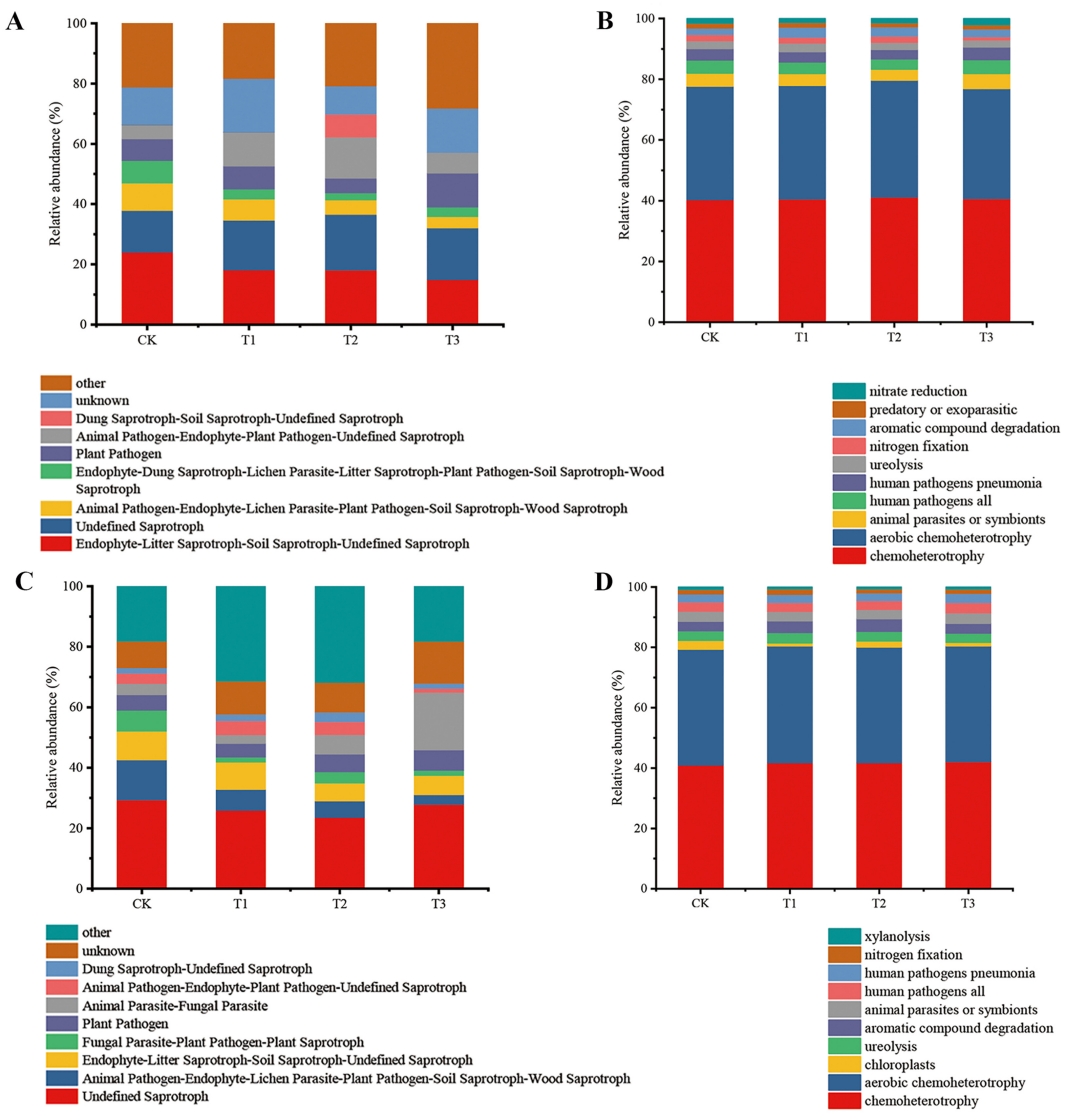
Functional prediction analysis of soil microbial communities. Panels **(A)** and **(B)** represent fungal and bacterial functional groups, respectively, in soil samples collected immediately after RSD treatment (0 d). Panels **(C)** and **(D)** represent fungal and bacterial functional groups, respectively, in soil samples collected at the tobacco vigorous growing stage (60 d post-transplanting).

FUNGuild functional prediction showed that RSD treatment significantly regulated fungal trophic functional composition. Immediately following RSD treatment, the relative abundance of the Plant Pathogen trophic mode was markedly lower in the T2 and T3 treatments (Figure 8A). Concurrently, the relative abundance of saprotrophic modes including Undefined Saprotroph and Dung Saprotroph-Soil Saprotroph-Undefined Saprotroph was increased. Symbiotrophic-saprotrophic modes including Endophyte-Litter Saprotroph-Soil Saprotroph-Undefined Saprotroph and Endophyte-Dung Saprotroph-Lichen Parasite-Litter Saprotroph-Soil Saprotroph-Wood Saprotroph were decreased. At 60 d, the Fungal Parasite-Plant Pathogen-Plant Saprotroph mode was significantly reduced in RSD treatments. The Plant Pathogen mode remained markedly lower (Figure 8C). The overall proportion of saprotrophic modes including Undefined Saprotroph and Dung Saprotroph-Undefined Saprotroph were increased. The Endophyte-Litter Saprotroph-Soil Saprotroph-Undefined Saprotroph mode was significantly reduced. These results demonstrated that RSD treatment could directly reduce the functional potential of soil pathogens and enhance organic matter decomposition functions. This effect was persistent.

FAPROTAX-based functional prediction for bacterial communities revealed that chemoheterotrophy and aerobic chemoheterotrophy were the dominant core functions across all soil samples (Figure 8B). Their combined relative abundance exceeded 75%. Immediately after RSD treatment, functional groups involved in soil nitrogen cycling were significantly altered in the T3 treatment. The relative abundance of bacteria associated with nitrate reduction significantly increased. Conversely, the relative abundances of bacteria associated with nitrogen fixation and ureolysis significantly decreased. No significant changes were observed in other functional groups. By 60 d, no significant differences in the functional composition of bacterial communities were detected among treatments (Figure 8D). This indicated that RSD treatment exerted significant short-term regulatory effects on bacterial functions, which gradually stabilized during the tobacco growth period.

### 3.7 Effects of RSD treatment on tobacco agronomic traits

RSD treatment combined with organic matter application promoted tobacco growth. At the topping stage, plant height, stem girth, and maximum leaf area in T2 and T3 were significantly higher than in CK (Table 3). Relative to CK, plant height in the T2 and T3 treatments increased by 0.9% and 1.6%, respectively. Maximum leaf area increased by 7.9% and 10.4%. T3 showed the greatest significant increases for all indicators. In contrast, T1 showed decreases of 3.1%, 0.8%, and 3.5% for height, girth, and maximum leaf area.

**Table 3:** Effects of RSD treatment combined with reduced chemical fertilizer on agronomic traits of tobacco at the topping stage.

Treatment	Effective leaf number	Height (cm)	Stem circumference (cm)	Maximum leaf area (cm^2^)
CK	19 a	109.70 ± 1.25 ab	9.47 ± 0.21 b	1,564.15 ± 28.63 bc
T1	19 a	106.25 ± 1.41 bc	9.39 ± 0.18 b	1,509.50 ± 26.81 c
T2	19 a	110.65 ± 1.18 a	10.56 ± 0.20 a	1,687.85 ± 31.27 ab
T3	19 a	111.45 ± 1.23 a	10.90 ± 0.29 a	1,726.70 ± 26.95 a

Values are means (*n* = 3). Different lowercase letters within the same column indicate significant differences among treatments at *P* < 0.05.

### 3.8 Effect of RSD treatment on tobacco disease incidence

The primary diseases observed in the experimental area were tobacco bacterial wilt and tobacco ordinary mosaic virus disease. The incidence and disease index of tobacco bacterial wilt was significantly lower in all RSD treatments compared to CK (Table 4). Relative to CK, the disease incidence in the T2 and T3 treatments was reduced by 60.00% and 93.33%, respectively. The disease index was reduced by 68.92% and 97.57%, respectively. This demonstrated that RSD treatment had significant control efficacy against tobacco bacterial wilt, with the T3 treatment being the most effective. RSD treatment also exhibited significant control efficacy against tobacco ordinary mosaic virus disease. Compared to CK, disease incidence in the T2 and T3 treatments was reduced by 64.71% and 76.47%, respectively, while the disease index was reduced by 55.67% and 83.40%. These results indicated that RSD treatment conferred resistance to tobacco ordinary mosaic virus disease, with the T3 treatment showing the optimal effect.

**Table 4:** Effects of RSD treatment on disease incidence in tobacco.

Treatment	TMV		PVY	
	Incidence (%)	Disease index	Incidence (%)	Disease index
CK	56.67 ± 0.03 a	10.58 ± 0.41 a	50.00 ± 0.06 a	14.96 ± 0.56 a
T1	23.33 ± 0.09 b	2.71 ± 0.95 c	43.33 ± 0.03 a	15.91 ± 0.34 a
T2	20.00 ± 0.00 b	4.69 ± 0.23 b	20.00 ± 0.00 b	4.65 ± 0.32 b
T3	13.33 ± 0.03 b	1.76 ± 0.34 c	3.33 ± 0.03 c	0.36 ± 0.36 c

Values are means ± standard error (*n* = 3). Different lowercase letters within the same column indicate significant differences among treatments at *P* < 0.05 (Duncan's multiple range test). Disease investigation was conducted according to the national standard GB/T 23222-2008. TMV, tobacco ordinary mosaic virus; PVY, Tobacco potato virus Y.

### 3.9 Effect of RSD treatment on chemical composition of tobacco leaves

RSD treatment significantly regulated the chemical composition and coordination of tobacco leaves. Compared with CK, the contents of total sugar, reducing sugar, starch, total nitrogen, and nicotine in T2 and T3 increased (Table 5). starch content in the T3 treatment was significantly increased by 57.1% relative to CK (*P* < 0.05). Chlorine content was significantly reduced by 17.4% and 40.6% in the T2 and T3 treatments, respectively (*P* < 0.05). Consequently, the potassium-to-chlorine ratio was significantly increased by 24.8% and 74.2% in these treatments (*P* < 0.05), indicating a marked improvement in tobacco leaf combustibility. The sugar-to-nicotine ratio for all treatments fell within the optimal range for high-quality tobacco (8–12). The nitrogen-to-nicotine ratio also conformed to the standard for high-quality tobacco (0.8–1.0). The overall chemical composition balance was satisfactory. In summary, the T3 treatment was most effective in reducing leaf chlorine content and increasing the potassium-to-chlorine ratio. The T2 treatment performed better in increasing leaf potassium content. No significant differences were observed between these two treatments for the remaining chemical components.

**Table 5:** Effects of RSD treatment on major chemical components of tobacco leaves.

Treatment	Total nitrogen (%)	Nicotine (%)	Total sugar (%)	Reducing sugar (%)	Starch (%)	NNR	SNR	KCR	Potassium (%)	Chlorine (%)
CK	2.10 ± 0.03 cb	3.58 ± 0.10 c	27.18 ± 0.97 b	19.89 ± 0.77 b	2.40 ± 0.13 c	0.59 ± 0.01 b	7.61 ± 0.46 ab	2.95 ± 0.18 c	2.03 ± 0.08 ab	0.69 ± 0.02 a
T1	2.20 ± 0.01 ab	3.29 ± 0.03 c	29.46 ± 0.51 ab	22.24 ± 0.30 a	3.17 ± 0.18 bc	0.62 ± 0.01 a	6.80 ± 0.21 b	3.94 ± 0.08 b	2.08 ± 0.03 ab	0.49 ± 0.01 bc
T2	2.19 ± 0.05 ab	3.71 ± 0.14 ab	27.07 ± 1.38 b	21.07 ± 1.02 ab	2.95 ± 0.48 bc	0.59 ± 0.01 ab	7.34 ± 0.56 ab	3.68 ± 0.13 bc	2.10 ± 0.08 a	0.57 ± 0.04 b
T3	2.22 ± 0.04 a	3.87 ± 0.04 a	28.22 ± 0.21 b	20.61 ± 0.12 ab	3.77 ± 0.28 b	0.57 ± 0.02 b	7.30 ± 0.05 ab	5.14 ± 0.45 a	2.07 ± 0.02 ab	0.41 ± 0.04 cd

Values are means ± standard error (*n* = 3). Different lowercase letters within the same column indicate significant differences among treatments at *P* < 0.05. Tobacco leaf samples were of the C3F grade. NNR, Nitrogen-nicotine ratio; SNR, Sugar-nicotine ratio; KCR, Potassium-chlorine ratio.

## 4 Discussion

### 4.1 Effects of RSD treatment on soil physicochemical properties

Soil acidification, organic matter decline, and nutrient imbalance induced by long-term continuous tobacco cropping are primary triggers of continuous cropping obstacles. These factors represent direct manifestations of soil ecosystem deterioration (Zhao et al., 2024). This study demonstrated that RSD treatment significantly increased soil pH and available nutrient contents. These improvement effects persisted through the middle and late stages of tobacco growth. The increase in soil nutrients was driven by the combination of direct organic material input and microbial mineralization (Zhan et al., 2024a). The application of distiller's grains and corn straw mixture directly augmented the soil organic matter pool. It also stimulated soil microbial activity, accelerating the transformation of organic nitrogen and phosphorus into available inorganic forms (Zhang et al., 2023). Improvements in soil pH were associated with the decomposition of organic acid intermediates and ammonia accumulation. Proton consumption during iron and manganese reduction also contributed to this shift. These processes are significant for alleviating acid stress in the acidic yellow soils of Bijie (Zhan et al., 2024b). Residual effects of RSD lasted until the tobacco maturation stage. From 50 to 140 days post-transplanting, soil nutrient contents in the T2 and T3 treatments remained significantly higher than in CK. This was attributed to RSD treatment improving soil aggregate structure and restoring nutrient adsorption and retention capacity (Yang et al., 2024). Refractory components in the organic material, such as cellulose and lignin, were not completely mineralized during the RSD treatment period. Subsequently, they served as a carbon source, continuously stimulating microbial activity and maintaining nutrient release (Li et al., 2026). Minimal differences between T2 and T3 suggest that 140 kg·per 667 m^2^ of organic material is sufficient for soil fertility needs.

### 4.2 Effects of RSD treatment on soil microbial community structure

Imbalances in microbial community structure and the enrichment of pathogens are central drivers of continuous cropping obstacles. The primary advantage of RSD technology is the directional reshaping of these degraded microbial communities (Duan et al., 2025; Fan et al., 2022). Our results revealed that RSD treatment differentially regulated soil fungal and bacterial communities. Fungal diversity and richness increased while bacterial diversity decreased despite an increase in richness. Significant increases in fungal Chao1 and Simpson indices were noted for T2 and T3. Conversely, the bacterial Simpson index decreased significantly. These changes reflect a selective filtering effect of the strong reductive environment on different microbial groups.

Alterations in diversity were driven by improved soils structure and the suppression of pathogens to reset ecological niches. RSD selectively enriched key fungal groups involved in organic matter decomposition (Yin et al., 2023). Ascomycota and Basidiomycota are the primary lignocellulose decomposers in soil, increased in relative abundance, facilitating rapid organic material degradation and nutrient release (Kalntremtziou et al., 2023; Yin et al., 2025). The inhibitory effect of RSD treatment on pathogenic fungi was particularly pronounced. The relative abundance of *Fusarium* decreased by 55.3%−68.1% compared with CK. *Gibberella* and *Phoma* showed similar trends. The genus *Fusarium* includes various causal agents of soilborne diseases. Its reduction is a direct cause for the lower incidence of tobacco bacterial wilt (Luo et al., 2023; Molnár et al., 2023).

Selective filtering was also observed in bacterial communities. Relative abundances of Acidobacteriota, *Chloroflexi*, and *Bacillus* increased while Proteobacteria decreased. Most Proteobacteria are aerobic bacteria such as *Sphingomonas*. The strong reductive environment directly inhibited these groups (Zhang et al., 2019). Most members of Acidobacteriota are acidophilic and participate in cellulose degradation and nitrogen cycling. Their increase correlated with improved pH and organic inputs (Chen et al., 2025; Liu et al., 2022). *Chloroflexi*, as facultative anaerobic photoautotrophs, can participate in carbon, nitrogen, and sulfur cycling. They may also promote plant growth and inhibit soilborne pathogens by producing hormones, solubilizing phosphate, and secreting siderophores (Nelkner et al., 2019). *Bacillus* is a well-recognized plant growth-promoting rhizobacterium. It promotes tobacco growth and suppresses soilborne diseases through various mechanisms, including secreting antimicrobial substances, inducing systemic resistance in plants, and solubilizing soil nutrients (Fan et al., 2023). The significant increase in *Bacillus* abundance was a key mechanism underlying RSD treatment's promotion of tobacco growth and enhancement of disease control (Zhu et al., 2024).

Exogenous organic amendment input was a core prerequisite for RSD to drive directional succession of the soil microbial community. Anaerobic treatment without organic material supplementation failed to yield consistently differentially abundant fungal or bacterial biomarkers. In contrast, the microbial communities in organic-amended RSD treatments exhibited a clear dynamic succession, characterized by pathogen suppression and beneficial microorganism enrichment. During the RSD remediation, saprotrophic decomposer groups dominated the community, facilitating mineralization of the added organic materials and inhibiting soilborne pathogens. The high organic input treatment (T3) led to significant enrichment of saprotrophic fungi such as *Peziza* and strictly anaerobic bacteria including *Anaerolineae*, which served as core functional groups driving rapid mineralization of the applied organic materials. The organic acids and reducing compounds generated by the metabolism of these taxa directly explained the mechanisms underlying pathogen inactivation, as well as the observed increases in soil pH and available nutrient contents. By the tobacco vigorous growth stage, the bacterial and fungal communities had shifted toward functional groups dominated by plant growth-promoting rhizobacteria and biocontrol agents. Within the fungal community, the T2 and T3 treatments led to the enrichment of saprotrophic taxa with potential biocontrol, including *Leucoagaricus* and *Myceliophthora*, respectively (Hu et al., 2022; Tássia et al., 2025). Within the bacterial community, T3 resulted in significant enrichment of plant growth-promoting and biocontrol bacteria such as *Ensifer* and *Fictibacillus*, while the T2 treatment enriched taxa involved in continuous organic matter decomposition, including *Bryobacter* and members of the family Chitinophagaceae (Chacón et al., 2022; Du et al., 2024; Montoya et al., 2025).

### 4.3 Correlation between microbial communities and soil physicochemical properties

The coupling between community succession and soil properties reveals the synergistic mechanisms of RSD restoration. Fungal genera like *Mortierella, Fusarium*, and *Tausonia* were negatively correlated with soil AN and SOM. RSD generates organic acids and ammonia which force a decline in these specific abundances. Concurrently, microbial decomposition of organic materials increases AN and SOM concentrations (Zhang et al., 2025). *Gibellulopsis* exhibited a highly significant positive correlation with pH, suggesting that the pH environment during RSD treatment was favorable for its growth. Regarding bacteria, *Gemmatimonadota* was significantly negatively correlated with soil AN and SOM. This might be due to the organic material degradation process during RSD inhibiting the proliferation of denitrifying bacteria within *Gemmatimonadota*. *Bacteroidota* positively correlated with pH, AN, and SOM. This group participates in the decomposition of residues and nitrogen mineralization to increase nutrient levels and regulate pH (Liu et al., 2023; Zhu et al., 2020).

### 4.4 Effects of RSD treatment on soil microbial functional changes

FUNGuild predictions showed that RSD significantly reduced plant pathogenic fungi while increasing saprotrophs. This effect lasted until the vigorous growth stage of tobacco. Relative abundances of plant pathogens in T2 and T3 were notably lower than CK. Saprotrophic functions increased significantly. Samples from the vigorous growth stage showed continued reductions in fungal parasites and plant pathogens. Functional remodeling confirms that RSD reshapes the microbial ecology to enhance disease suppression (Ali et al., 2023; Zhao et al., 2025).

FAPROTAX results indicated that chemoheterotrophy and aerobic chemoheterotrophy were dominant bacterial functions. Their combined relative abundance exceeded 75%. This reflects the heterotrophic metabolic characteristics of dryland tobacco soil bacteria using organic carbon. Analysis of nitrogen-cycling functions revealed that the high-rate organic material RSD treatment (T3) significantly altered the relative abundance of nitrogen-cycling functional groups. Specifically, the abundance of nitrate reduction functions significantly increased, while those of nitrogen fixation and ureolysis significantly decreased. This directional change in nitrogen-cycling functions could enhance available nitrogen supply by regulating soil nitrogen transformation processes. Furthermore, volatile substances like ammonia produced through anaerobic metabolism could contribute to inactivating soilborne pathogens. This effect was closely linked to the enrichment of nitrogen-cycling-related functional bacteria such as *Bacillus* (Di Gioia et al., 2017; Li et al., 2022).

### 4.5 RSD treatment reshaped co-occurrence networks

Variations in microbial network topology reflect underlying shifts in community stability and interaction intensity under external management regimes (Liu et al., 2020). In this study, fungal and bacterial networks exhibited divergent responses to RSD treatment across sampling stages. Despite RSD enhancing fungal alpha diversity (Table 2), co-occurrence parameters including average degree, density, and modularity remained largely invariant between CK and T3. This decoupling between diversity and network architecture suggests that RSD promoted taxonomic richness without disrupting the pre-existing interaction framework (de Vries et al., 2018). In contrast, bacterial networks underwent substantial structural remodeling. RSD significantly enhanced network complexity, elevating edge counts, density, and average degree at both the initial stage and 60 days after transplanting. Lower modularity implies fewer ecological barriers and stronger cross-module interactions, facilitating efficient material cycling and signal transduction (Gu et al., 2020). This architectural shift was further supported by the enrichment of plant growth-promoting taxa (e.g., *Bacillus, Arthrobacter*) and the concomitant suppression of pathogens (e.g., *Fusarium, Gibberella*). The treatment preferentially amplified bacterial network integration while preserving the inherent stability of fungal networks. This coordinated adjustment fortifies the overall integrity and stress resistance of the soil ecosystem.

### 4.6 Effects of RSD treatment on agronomic traits and disease incidence

Long-term continuous cropping significantly inhibits crop vegetative growth, leading to deteriorated agronomic traits and hindered dry matter accumulation. This ultimately results in yield reduction, directly reflecting productivity decline in continuously cropped tobacco soils (Gao et al., 2021; Zeeshan Ul Haq et al., 2023). RSD treatments with organic materials (T2 and T3) significantly increased plant height, stem girth, and maximum leaf area. In contrast, anaerobic treatment without organic matter (T1) combined with reduced fertilizer inhibited growth. This clarified that organic material is a prerequisite for ensuring normal tobacco growth within an RSD system under reduced fertilizer application. The growth-promoting effect of RSD treatment originated from its amelioration of soil acidification and sustained supply of available nutrients in continuous cropping soil. This alleviated nutrient limitation factors (Butler et al., 2014). Enriched rhizosphere growth-promoting bacteria, such as *Bacillus*, could also directly stimulate tobacco growth by secreting plant growth regulators and activating rhizosphere nutrients. Concurrently, the significant increase in maximum leaf area expanded the plant's photosynthetic capture area. This provided a material foundation for dry matter accumulation and yield formation.

Tobacco diseases are aggravated by continuous cropping. This reduces leaf quality and yield (Jia et al., 2024). T2 and T3 treatments showed significant control of tobacco bacterial wilt and tobacco mosaic virus (TMV). Control efficacy increased with the amount of organic material applied. These results indicated that RSD treatment significantly enhanced tobacco resistance to TMV and bacterial wilt. The reduced relative abundance of pathogenic fungi like *Fusarium* directly decreased infection pressure. Furthermore, the beneficial microbial community reshaped by RSD could inhibit pathogen recolonization through niche competition and secretion of antimicrobial substances. This further consolidated disease control effectiveness (Zhan et al., 2021). As a viral disease, the decreased incidence of TMV is difficult to attribute to direct soil microbial action on the virus. It is more likely attributable to plant systemic resistance induced by RSD treatment (Hernández-Muñiz et al., 2024). Enriched rhizosphere growth-promoting bacteria, such as *Bacillus*, can induce systemic resistance in tobacco plants. This confers broad-spectrum resistance against various pathogens, including viruses and fungi (Haddoudi et al., 2021). The improvement in tobacco vegetative growth and the reduction in disease pressure formed a positive synergy (Castillo-Díaz et al., 2023).

### 4.7 Effects of RSD treatment on tobacco chemical composition

RSD treatment optimized the chemical composition and coordination of tobacco leaves. Sugar content increased to enhance the sweetness of the smoke. Starch content rose with higher organic material application to improve taste. T3 performed best in balancing starch accumulation and sugar content. Coordination indices are essential for evaluating tobacco quality (Wu et al., 2024b). Sugar-to-nicotine ratios in all treatments were within the suitable range for high-quality tobacco (Chen et al., 2021). The potassium-to-chlorine ratio was significantly higher than in CK, indicating markedly improved tobacco leaf combustibility. Overall, the T3 treatment demonstrated the best performance in maintaining an appropriate nitrogen-to-nicotine ratio, optimizing the sugar-to-nicotine ratio, and increasing the potassium-to-chlorine ratio.

## 5 Conclusion

This study demonstrated that reductive soil disinfestation combined with organic material substitution can effectively alleviate continuous cropping obstacles in upland tobacco systems of karst regions. The treatment with 280 kg per 667 m^2^ organic material and a 20% reduction in chemical fertilizer (T3) showed the strongest effects, as evidenced by significant suppression of soilborne pathogens, enrichment of beneficial bacteria, and substantial reductions in disease incidence. RSD treatment also improved soil pH, available nutrients, and leaf chemical composition, particularly the potassium-to-chlorine ratio. These findings provide a scientific basis for green management of continuous cropping obstacles in karst mountainous tobacco-growing areas.

## Data Availability

The original contributions presented in the study are included in the article/Supplementary material, further inquiries can be directed to the corresponding author.
