# Supplementary material for: Reductive soil disinfestation alleviates continuous cropping obstacles in tobacco by reshaping microbial communities and improving soil properties in karst regions
Source: Front Microbiol. 2026 May 28;17:1841207. doi: 10.3389/fmicb.2026.1841207 (PMC13253632; doi:10.3389/fmicb.2026.1841207)
Supplement: Supplementary file 1 [file Data_Sheet_1.pdf]

SUPPLEMENTARY FIGURE S1. Linear discriminant analysis Effect Size (LEfSe) analysis of soil microbial communities. Panels (A) and (B) represent fungal and bacterial communities, respectively, in soil samples collected immediately after RSD treatment (0 d). Panels (C) and (D) represent fungal and bacterial communities, respectively, in soil samples collected at the tobacco vigorous growing stage (60 d post-transplanting). Only taxa with a linear discriminant analysis (LDA) score greater than 2.0 ($P < 0.05$) are shown.
