# Supplementary figures and images for: Reductive soil disinfestation alleviates continuous cropping obstacles in tobacco by reshaping microbial communities and improving soil properties in karst regions
Source: Front Microbiol. 2026 May 28;17:1841207. doi: 10.3389/fmicb.2026.1841207 (PMC13253632; doi:10.3389/fmicb.2026.1841207)

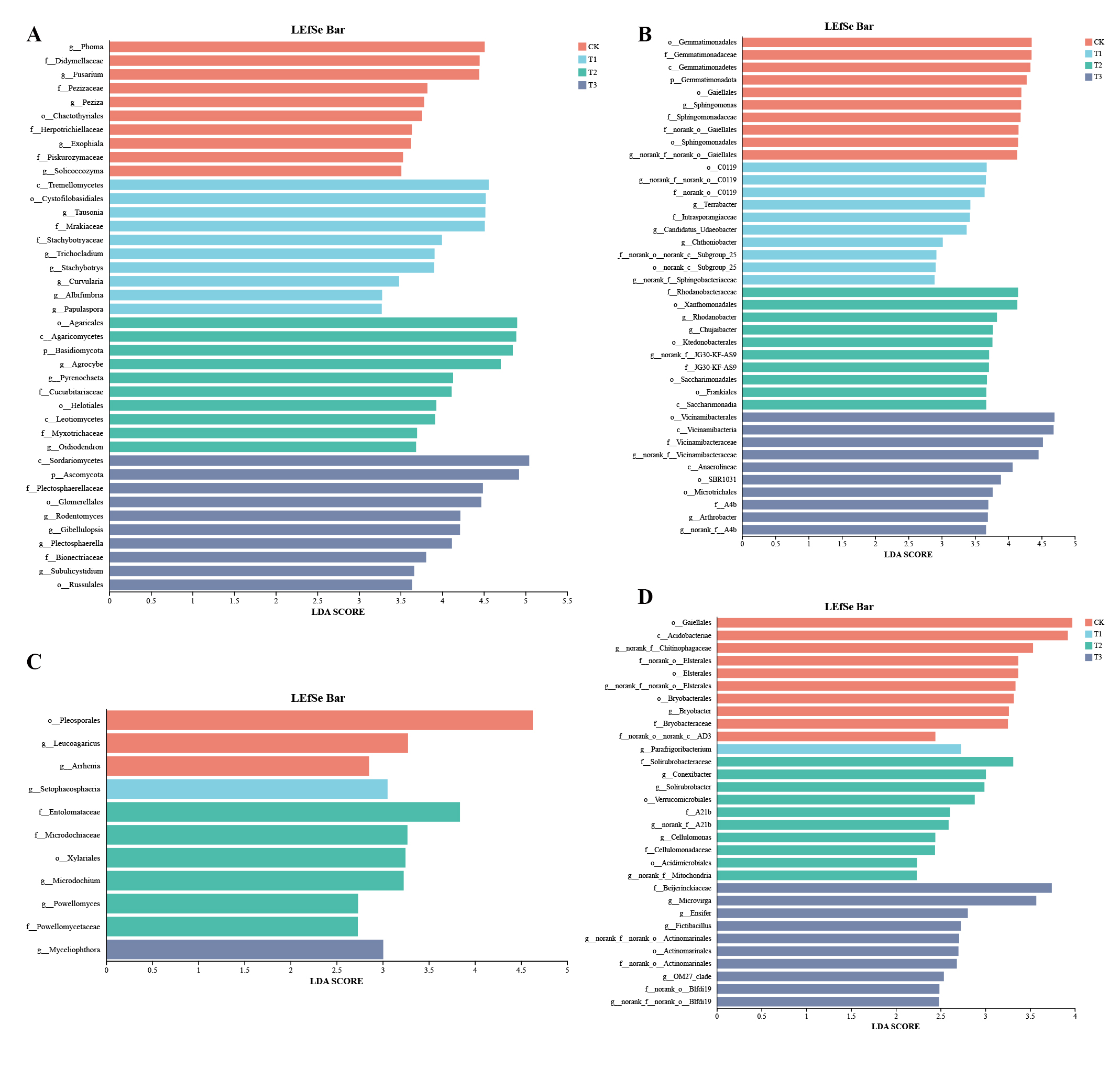

Supplement: Supplementary file 2 [file Image_1.jpeg]
